# Type 3A free anterolateral thigh chimeric flap based on the Zunyi classification for reconstructing diabetic foot wounds

**DOI:** 10.3389/fsurg.2025.1679998

**Published:** 2025-11-04

**Authors:** Tianhua Zhang, Xiangkui Wu, Lingli Jiang, Shun’e Xiao, Hai Li, Wei Chen, Shusen Chang, Bihua Wu, Zairong Wei, Chengliang Deng

**Affiliations:** Department of Burns and Plastic Surgery, Affiliated Hospital of Zunyi Medical University, Zunyi, China

**Keywords:** diabetic foot wounds (DFWs), anterolateral thigh flap (ALT), Zunyi classification, Type 3A chimeric flap, limb salvage, microsurgical reconstruction, multi-tissue defects, functional outcome

## Abstract

**Background:**

Diabetic foot wounds (DFWs) often present with multilayered tissue loss involving skin, fascia, muscle, and neurovascular structures, posing substantial reconstructive challenges. The Zunyi Classification provides a function-oriented framework for chimeric anterolateral thigh (ALT) flap design. This study aimed to evaluate the clinical efficacy of the Zunyi Type 3A chimeric ALT flap in the reconstruction of complex DFWs.

**Methods:**

A retrospective cohort study was carried out involving 28 patients with Wagner grade 2–4 DFWs who underwent reconstruction using a Zunyi Type 3A chimeric ALT flap between June 2021 and June 2024. All flaps were designed based on a single perforator without skin paddle segmentation. Flap composition was tailored to defect characteristics, incorporating combinations of skin, fascia lata, muscle, and sensory nerve. Primary outcomes included flap survival, complication rate, donor-site morbidity, and long-term functional outcome.

**Results:**

Among the 28 patients with Wagner grade 2–4 DFWs, the most common flap composition was skin + fascia lata + muscle (S + F + M), applied in 17 cases (60.7%). This was followed by skin + muscle + sensory nerve (S + M + N) in 6 cases (21.4%) and skin + fascia lata + sensory nerve (S + F + N) in 5 cases (17.9%). Flap survival was excellent, with 96.4% demonstrating complete viability; one case of partial distal necrosis was managed successfully with debridement and skin grafting. No donor-site complications were observed. During a median follow-up of 13 months (range: 3–30), none of the patients had ulcer recurrence, major infection, or limb-threatening events occurred. Most patients regained satisfactory ambulatory function while preserving limb integrity.

**Conclusions:**

The Zunyi Type 3A chimeric ALT flap offers a reliable, adaptable solution for limb salvage in patients with advanced DFWs. Its capacity to incorporate multiple tissue types in a single, unsegmented design offers effective coverage, structural support, and long-term durability. These findings support broader clinical application of the Zunyi Classification in personalized reconstructive strategies for complex DFWs.

## Highlights

• The Zunyi Type 3A chimeric ALT flap enables anatomically layered, unsegmented reconstruction of DFWs by incorporating skin, fascia, muscle, and sensory nerve in a single-perforator design, offering structural support and sensory restoration.

• This retrospective study of 28 patients showed a 96.4% flap survival rate with no donor-site complications and no ulcer recurrence, underscoring the clinical reliability and long-term durability of the Zunyi Type 3A flap in complex limb salvage.

## 1 Introduction

Diabetic foot wounds (DFWs) remain a formidable reconstructive challenge due to their chronicity, deep tissue involvement, and high risk of infection and limb loss (1–3). These complex wounds often require more than just skin coverage—they demand the repair of deeper anatomical structures such as muscle, tendon, and bone (4, 5). Traditional flap options frequently fall short in addressing these multidimensional deficits.

The anterolateral thigh (ALT) flap has become a mainstay in microsurgical reconstruction, offering a reliable skin paddle with the potential for multiple tissue components (6, 7). With the evolution of chimeric flap techniques, it is now possible to harvest various tissue types from a single vascular source, enabling personalized three-dimensional reconstruction (8, 9). However, most existing classification systems for chimeric flaps are grounded in vascular anatomy rather than surgical practicality. To bridge this gap, the Zunyi Classification was introduced—a function-oriented stratification system based on tissue composition and reconstructive complexity for free ALT chimeric flaps. Within this framework, the Type 3A flap—comprising skin, fascia, nerve, muscle, and optionally bone in an undivided configuration—is specifically designed to address wounds requiring both surface coverage and deep structural restoration (10).

This study aims to evaluate the clinical efficacy of the Zunyi Type 3A free ALT chimeric flap in the reconstruction of DFWs. By analyzing flap survival, complication rates, healing time, and functional outcomes, we seek to validate the Zunyi Classification as a practical and effective framework for guiding flap selection and improving limb salvage in patients with advanced DFWs.

## 2 Materials and methods

This was a single-center, retrospective cohort study conducted in the Department of Burn and Plastic Surgery at the Affiliated Hospital of Zunyi Medical University. This study was approved from the institutional review board of the Affiliated Hospital of Zunyi Medical University (KLL-2025-043). A total of 28 patients with DFWs who underwent reconstruction with a Type 3A chimeric anterolateral thigh (ALT) flap between June 2021 and June 2024 were enrolled. The study protocol adhered to the principles of the Declaration of Helsinki. Informed consent was obtained from all patients and their attending surgeons prior to surgery. Clinical and operative data were collected through retrospective review of medical records. Variables included patient demographics (e.g., sex), defect location and dimensions, flap size and composition, postoperative complications, and follow-up outcomes (Table 1).

**Table 1:** Patient characteristics.

Case	Sex	Wagner grade	Defect size (cm)	Flap size (cm)	Composition	Complications	Follow-up (mo)
1	M	3	10 × 6	12 × 7	S + M + N	None	12
2	F	2	9 × 5	11 × 6	S + F + M	None	8
3	M	4	16 × 7	18 × 8	S + F + M	None	6
4	M	3	14 × 7	16 × 8	S + F + M	None	14
5	F	3	11 × 6	13 × 7	S + F + M	None	10
6	M	4	18 × 7	20 × 8	S + F + M	None	24
7	M	2	8 × 5	10 × 6	S + F + N	None	18
8	M	3	12 × 6	14 × 7	S + M + N	None	9
9	M	3	13 × 5	15 × 6	S + F + N	None	11
10	M	4	17 × 8	19 × 9	S + F + M	Partial necrosis	16
11	F	3	10 × 6	12 × 7	S + M + N	None	7
12	M	2	9 × 5	11 × 6	S + F + M	None	5
13	F	4	15 × 8	17 × 9	S + M + N	None	20
14	F	3	11 × 6	13 × 7	S + M + N	None	13
15	M	3	12 × 7	14 × 8	S + F + M	None	9
16	M	4	19 × 9	21 × 10	S + F + M	None	22
17	F	2	8 × 4	10 × 5	S + F + M	None	4
18	M	3	13 × 7	15 × 8	S + M + N	None	12
19	M	3	14 × 6	16 × 7	S + F + M	None	15
20	M	4	16 × 8	18 × 9	S + F + M	None	21
21	M	3	11 × 5	13 × 6	S + F + M	None	8
22	M	2	9 × 5	11 × 6	S + F + M	None	6
23	F	3	12 × 6	14 × 7	S + F + N	None	10
24	M	4	18 × 9	20 × 10	S + F + M	None	25
25	M	3	10 × 6	12 × 7	S + F + M	None	11
26	F	3	13 × 6	15 × 7	S + F + N	None	14
27	M	4	17 × 8	19 × 9	S + F + M	None	30
28	F	2	8 × 4	10 × 5	S + F + N	None	3

All flaps were harvested based on a single perforator. In the Zunyi Type 3A configuration, multiple tissue components—including skin, fascia, nerve, and muscle—were incorporated as needed without segmentation of the skin paddle, allowing anatomically layered reconstruction with simplified vascular anastomosis.

Composition S, skin paddle; F, fascia lata; M, muscle; N, sensory nerve branch.

### 2.1 Inclusion and exclusion criteria

Inclusion criteria were as follows: (1) Wagner grade 2–4 ulcers with well-controlled blood glucose and infection; (2) patients in stable general condition and able to tolerate anesthesia and microsurgery; (3) a well-prepared wound bed, either covered with healthy granulation tissue following thorough debridement or with a fresh, gelatinous induced membrane after temporary coverage with antibiotic-loaded bone cement; and (4) preoperative imaging confirming adequate distal limb perfusion—either by computed tomography angiography (CTA) showing patent major vessels, an ankle–brachial index (ABI) greater than 1.00, or peak arterial flow velocity ≥40 cm/s as measured by Doppler ultrasonography. (5) reconstruction performed using a Type 3A chimeric ALT flap as defined by the Zunyi Classification.

Exclusion criteria included: (1) severe systemic comorbidities rendering the patient unfit for anesthesia or major surgery; (2) proximal occlusion of major recipient vessels with no available distal target for vascular anastomosis; (3) end-stage diabetic nephropathy; (4) chronic renal failure requiring regular dialysis; (5) ongoing use of immunosuppressive agents; and (6) recipient site arterial peak flow velocity <20 cm/s.

### 2.2 Preoperative evaluation and flap classification

According to the Zunyi Classification, a Type 3A chimeric flap is defined as an unsegmented anterolateral thigh flap composed of three distinct tissue components—skin, nerve, fascia lata, muscle—based on a single vascular pedicle. Preoperative assessment included color Doppler ultrasonography to identify suitable perforators and to evaluate the vascular anatomy of the donor site.

### 2.3 Preoperative optimization and risk assessment

All patients received standardized preoperative education, including smoking cessation for a minimum of two weeks prior to surgery. The evaluation was carried out in two stages:

First-stage assessment included comprehensive physical examination, detailed inspection of DFWs, and measurement of the ankle–brachial index (ABI) on the affected limb, all of which were >1.00. Lower extremity arterial patency was evaluated via computed tomography angiography (CTA). In addition, cardiac, cerebral, and renal function were assessed to ensure systemic stability and surgical tolerance.

Second-stage assessment was performed prior to flap transplantation and included Doppler ultrasonography to measure the peak systolic velocity (PSV) of recipient arteries. In cases where recipient arteries were occluded or had a PSV ≤30 cm/s, vascular surgery consultation and intervention were initiated. Four patients in this cohort underwent preoperative percutaneous transluminal angioplasty (PTA) to improve arterial flow, achieving a PSV ≥40 cm/s to ensure adequate flap perfusion.

To optimize perioperative physiological status, patients received transfusions of ABO-compatible packed red blood cells and human serum albumin to maintain hemoglobin ≥90 g/L and serum albumin ≥30 g/L. Electrolytes and other metabolic parameters were corrected as needed. Glycemic control was achieved using an insulin pump to maintain fasting blood glucose levels within the range of 3.9–8.0 mmol/L.

### 2.4 Surgical technique

All procedures were performed under general anesthesia with patients in the supine position. Preoperative perforator mapping was carried out using the modified “Three-Vertical and Five-Horizontal” method to identify dominant cutaneous perforators from the descending branch of the lateral circumflex femoral artery. An incision was made sharply along the lateral border of the planned flap. Dissection was carried out in the superficial layer of the deep fascia while carefully preserving the lateral femoral cutaneous nerve and its accompanying nutrient vessels. Perforators were carefully traced through the subcutaneous tissue down to the level of the fascia lata. The fascia was incised to expose the perforator's course, type, and origin, with careful identification and preservation of the main vascular pedicle. The flap was harvested using the “meeting-in-the-middle” technique, to ensure the integrity of each chimeric component. Depending on the recipient site requirements, additional tissue components such as muscle, or fascia were enrolled. The dimensions and composition of each component were individualized according to defect characteristics, including the need for dead space obliteration, joint capsule reconstruction, or tendon coverage. Once the flap was elevated, microvascular anastomosis was performed under a surgical microscope, connecting the flap pedicle to recipient vessels. Donor sites were closed direct closure or covered with split-thickness skin grafts when primary closure was not feasible.

### 2.5 Postoperative management

Postoperatively, all patients were strictly confined to bed rest and advised to abstain from smoking. Fasting blood glucose levels were continuously monitored and maintained within the target range of 3.9–8.0 mmol/L using insulin pump therapy. Standard postoperative protocols included warming, vasodilation therapy, anticoagulation, prophylactic antibiotics, antispasmodics, and analgesia. Flap monitoring was performed at regular intervals, including assessments of skin color, temperature, and capillary refill to evaluate perfusion status. Rubber strip drains placed beneath the flap were removed on postoperative day 2. If the flap showed stable viability with no signs of vascular compromise or infection, patients were discharged between postoperative days 7 and 10. Sutures were removed approximately two weeks after surgery.

## 3 Results

**Figure 1:**
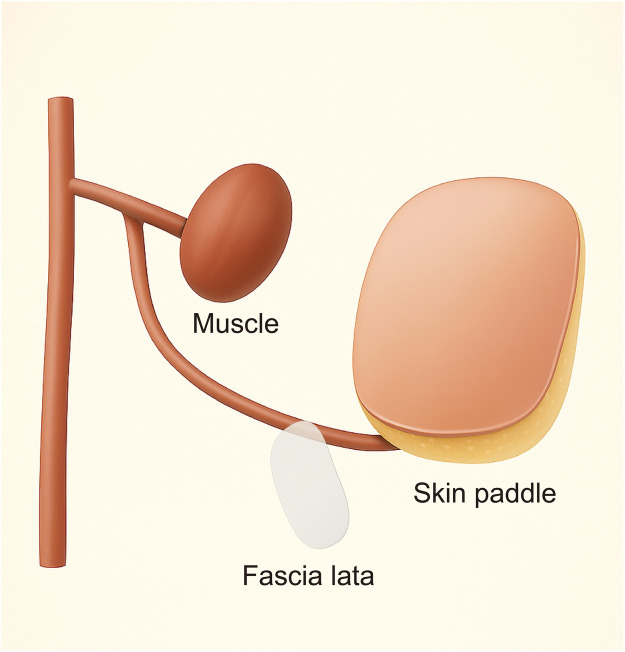
Schematic illustration of the Zunyi Type 3A chimeric anterolateral thigh (ALT) flap composed of skin paddle, fascia lata, and muscle (S + F + M).

A total of 28 patients with Wagner grade 2–4 DFWs underwent soft tissue reconstruction using the Zunyi Type 3A chimeric anterolateral thigh (ALT) flap. All flaps were harvested based on a single musculocutaneous perforator, without segmentation of the skin paddle. Flap design was tailored according to individual defect characteristics, incorporating various combinations of skin, fascia lata, muscle, and sensory nerve components. The three most frequent flap compositions were as follows: Skin + fascia lata + muscle (S + F + M) in 17 cases (60.7%) as Figure 1, Skin + muscle + sensory nerve (S + M + N) in 6 cases (21.4%). Skin + fascia lata + sensory nerve (S + F + N) in 5 cases (17.6%), Flap survival was excellent, with 27 of 28 flaps demonstrating complete viability. One patient experienced partial distal flap necrosis, which was managed successfully with surgical debridement and split-thickness skin grafting. All donor sites healed primarily, and no donor-site complications such as hematoma, infection, or functional morbidity were recorded.

The follow-up period ranged from 3–30 months (median: 13 months). Throughout this period, none of the patients had ulcer recurrence, major infection, or limb-threatening events were observed. All reconstructed wounds remained stable. Most patients regained satisfactory ambulatory function, while preserving limb integrity and no evidence of late flap failure or deterioration in functional outcomes. These findings highlight the clinical reliability, structural adaptability, and long-term durability of the Zunyi Type 3A chimeric ALT flap in the treatment of complex, multilayered defects in DFWs reconstruction.

## 4 Case reports

### 4.1 Case 3

**Figure 2:**
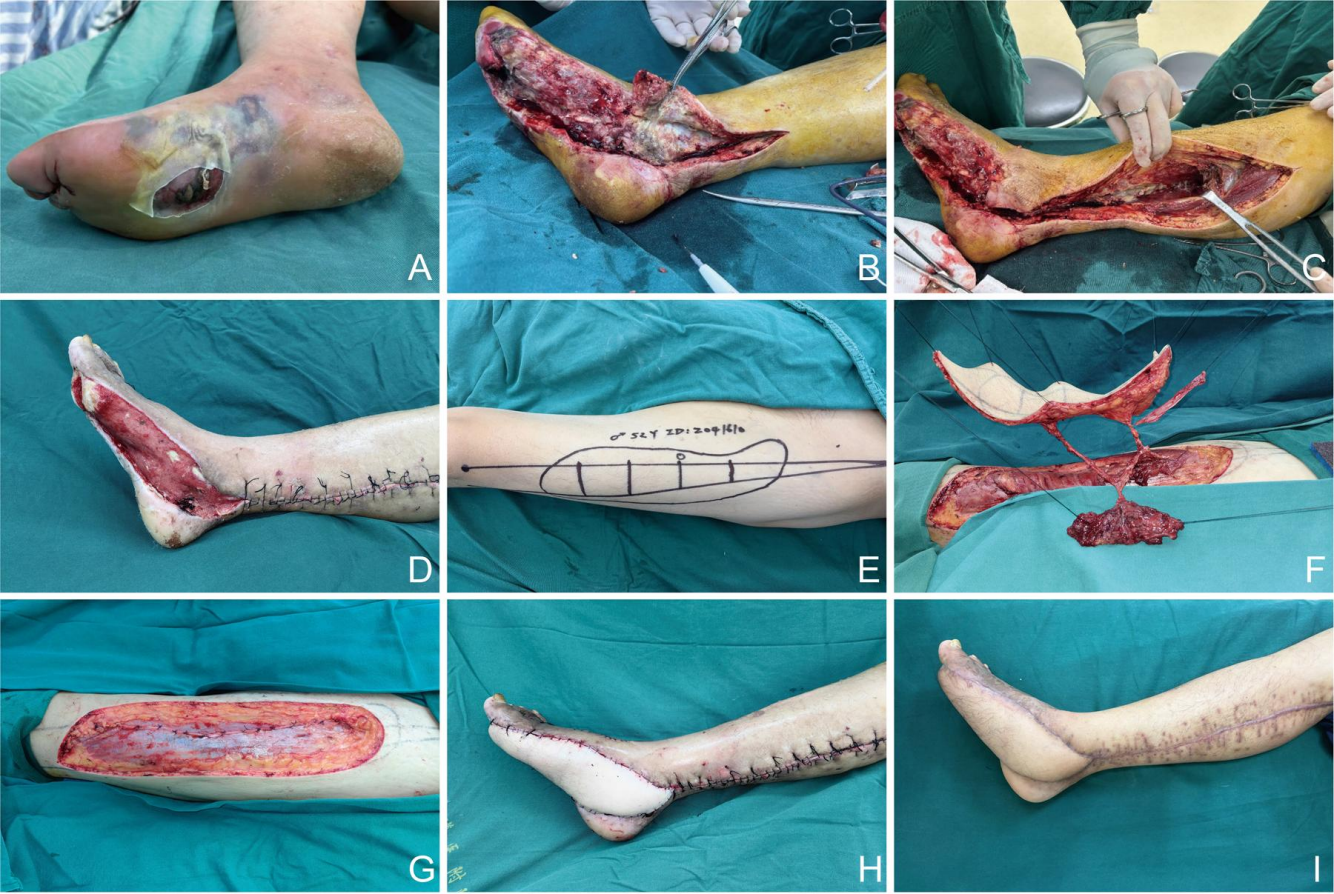
Reconstruction of a DFWs in a 52-year-old male using a Zunyi Type 3A chimeric ALT flap. **(A)** Preoperative foot with erythema, swelling, and purulent exudate. **(B,C)** Intraoperative debridement and fasciotomy exposing necrotic tissue. **(D)** Post-debridement defect with exposed joint capsule and tendons. **(E)** Flap design (18 × 8 cm) on the right thigh. **(F)** Harvested flap composed of skin, fascia, and muscle (S + F + M). **(G)** Donor site closed with complete fascia lata repair. **(H)** Flap inset with end-to-end anastomosis to the posterior tibial artery. **(I)** Six-month follow-up showing stable coverage and no ulcer recurrence.

A 52-year-old male patient with an 8-year history of type 2 diabetes mellitus presented with a Wagner grade 4 DFWs. On admission, physical examination showed marked erythema and elevated local temperature of the right foot. The dorsalis pedis artery pulse was weak, and sensory testing showed hypoesthesia in both dorsal and plantar aspects of the foot at areas of intact skin. Purulent exudate was observed upon compression (Figure 2A). Emergency debridement and fasciotomy of the right lower leg were performed (Figures 2B,C). Following radical debridement, a large soft tissue defect involving the medial ankle joint and dorsum of the foot was exposed, with evident exposure of the joint capsule and tendons (Figure 2D). A chimeric anterolateral thigh (ALT) flap, classified as Zunyi Type 3A, was harvested from the right thigh, measuring 18  ×  8 cm in size (Figure 2E). The flap composition included skin, fascia, and muscle (S + F + M) (Figure 2F). During flap harvesting, the fascia lata at the donor site was carefully sutured to restore continuity (Figure 2G). The vascular pedicle of the ALT flap was anastomosed end-to-end to the posterior tibial artery (Figure 2H). The flap survived completely without complications. At 6 months postoperatively, the flap exhibited satisfactory contour, texture, and durability. No new ulceration was observed in either the reconstructed limb or the contralateral limb (Figure 2I).

## 5 Discussion

DFWs present complex reconstructive challenges due to their multifactorial pathophysiology. Beyond the well-recognized triad of soft tissue deficits, deep infection, and sensory neuropathy, these wounds often exhibit impaired angiogenesis, microvascular thrombosis, and biofilm-laden chronic infections (11, 12). This leads to poor granulation, delayed healing, and high susceptibility to secondary ulceration. The affected areas frequently involve exposure of deep structures such as tendons, joint capsules, and bone, particularly in weight-bearing regions (13, 14). Moreover, autonomic dysfunction contributes to dry, inelastic skin prone to fissures, while motor neuropathy alters foot biomechanics, increasing localized pressure and shear stress. These combined factors create a biologically hostile and mechanically unstable wound environment. Therefore, optimal reconstruction must address both anatomical restoration and physiological compensation. This includes robust skin coverage, effective dead space obliteration, infection control, and—when feasible—restoration of protective sensation (15). In high-risk patients, the reconstructive strategy must also minimize donor-site morbidity and withstand long-term mechanical stress.

The Type 3A anterolateral thigh (ALT) chimeric flap is particularly well suited for addressing the multifaceted deficits seen in DFWs (10). These wounds often present with extensive soft tissue loss, exposure of deep structures, persistent infection, and neuropathy-related sensory deficits—challenges that demand layered, vascularized, and functionally tailored reconstruction (16, 17). The 3A flap meets these demands through its composite design. The undivided skin paddle offers robust coverage and can be harvested with cutaneous nerves to restore partial protective sensation, which is essential for preventing recurrence in neuropathic patients (18, 19). The muscle component fills dead space, improves local vascularity, and assists in bacterial clearance, which is particularly valuable in cases of deep abscess or osteomyelitis (20, 21). Fascia lata provides mechanical reinforcement, aiding in tendon or joint capsule repair, especially in weight-bearing regions. This anatomically and functionally integrated design enables precise, multi-tissue reconstruction tailored to each patient's defect. The flap's reliable perfusion—even in ischemic and infected fields—combined with its adaptability, makes it a powerful tool for limb salvage. In our series, the 3A flap showed excellent survival rates, reduced recurrence, and good long-term outcomes, underscoring its clinical superiority in complex DFWs reconstruction.

The present study shows that the Zunyi Type 3A chimeric anterolateral thigh (ALT) flap offers a reliable and versatile solution for complex soft tissue reconstruction in patients with Wagner grade 2–4 DFWs. By incorporating varying combinations of skin, fascia lata, muscle, and sensory nerve components, the flap can be precisely tailored to match the three-dimensional requirements of composite defects (22, 23). Notably, the S + F + M configuration was the most commonly utilized, reflecting the frequent need for structural support, dead space obliteration, and robust vascularized coverage in this patient population. Flap survival was high, with a 96.4% complete viability rate, and only one case of partial necrosis, which was effectively salvaged with minor revision procedures. Importantly, all donor sites healed uneventfully, with no complications related to hematoma, infection, or donor-site morbidity, underscoring the safety of this technique. During follow-up, no instances of flap breakdown, ulcer recurrence, or progressive limb deterioration were noted, supporting the long-term durability and functional integration of the flap.

These findings align with prior studies emphasizing the importance of chimeric flap designs in diabetic limb salvage, particularly when addressing multi-layer defects involving tendons, joints, and neurovascular structures (24). The inclusion of fascia lata contributes to tensile strength and contour stability, while the addition of muscle or nerve components addresses dead space management and sensory restoration, respectively (25). Collectively, the data support the clinical utility of the Zunyi Type 3A ALT flap as a reliable microsurgical option for limb preservation in complex DFWs reconstruction.

Compared to Type II flaps, which typically comprise skin and a single additional component (such as fascia or muscle), the Type 3A flap offers greater versatility for reconstructing the multilayered defects frequently seen in DFWs. In contrast, Type 3B flaps often require segmentation of the skin paddle and rely on two or more independent perforators, increasing surgical complexity, ischemic risk, and technical demands. The Type 3A flap, by incorporating multiple tissue elements—skin, fascia, muscle, and potentially sensory nerve—in a single, undivided unit based on one reliable perforator, achieves a favorable balance between reconstructive efficacy and surgical safety. This design minimizes intraoperative difficulty, reduces donor site morbidity, and preserves vascular integrity. Clinically, it results in fewer complications, accelerated recovery, and wider applicability, making it a highly suitable and efficient option for limb salvage in patients with advanced DFWs.

Based on our experience, successful flap harvest relies on accurate perforator mapping using Doppler ultrasound and the “Three-Vertical and Five-Horizontal” surface landmark technique. Preservation of the lateral femoral cutaneous nerve and intermuscular perforators is crucial for flap survival. The “meeting-in-the-middle” technique facilitates coordinated dissection, minimizes donor site bleeding, and reduces nerve injury. During anastomosis, end-to-side arterial connections are preferred to maintain distal limb perfusion and reduce the risk of spasm or thrombosis.

This study is limited by its single-center, retrospective design and relatively small sample size, which may introduce selection bias. The follow-up duration ranged from 3–30 months, and long-term functional assessments were not standardized. Additionally, successful application of the 3A flap requires a high level of surgical expertise in perforator identification and vascular dissection, potentially limiting its use in lower-resource settings or institutions lacking microsurgical experience.

The Zunyi Classification has the potential to be adopted as a universal system for ALT flap design, especially for staged or sequential reconstruction in complex wound management. Multicenter, prospective studies and standardized outcome databases are warranted to validate the efficacy and safety of each flap subtype across various clinical indications and patient populations.

## 6 Conclusion

The Zunyi Type 3A chimeric ALT flap provides an effective and versatile solution for DFWs reconstruction, integrating skin, fascia, nerve, muscle to address complex, multilayered defects. Its excellent survival rate, low complication profile, and good long-term outcomes highlight its value in limb salvage. The Zunyi Classification offers a practical, function-oriented framework for personalized flap design, meriting broader clinical adoption and further multicenter validation.

## Data Availability

The original contributions presented in the study are included in the article/supplementary material, further inquiries can be directed to the corresponding author.
